# To do or not to do—balancing governance and professional autonomy to abandon low-value practices: a study protocol

**DOI:** 10.1186/s13012-019-0919-x

**Published:** 2019-07-08

**Authors:** Henna Hasson, Per Nilsen, Hanna Augustsson, Sara Ingvarsson, Sara Korlén, Ulrica von Thiele Schwarz

**Affiliations:** 1 Procome research group, Department of Learning, Informatics, Management and Ethics, Medical Management Centre, Karolinska Institutet, SE 171 77 Stockholm, Sweden; 2 Unit for Implementation and Evaluation, Center for Epidemiology and Community Medicine (CES), Stockholm County Council, SE 171 29 Stockholm, Sweden; 3 Department of Medical and Health Sciences, Division of Community Medicine, Linköping University, Linköping, Sweden; 4 School of Health, Care and Social Welfare, Mälardalen University, Box 883, 721 23 Västerås, Sweden

**Keywords:** Governance, De-implementation, Low-value practices, Policy, Ineffective, Abandon, De-adoption, Decrease use

## Abstract

**Background:**

Many interventions used in health care lack evidence of effectiveness and may be unnecessary or even cause harm, and should therefore be de-implemented. Lists of such ineffective, low-value practices are common, but these lists have little chance of leading to improvements without sufficient knowledge regarding how de-implementation can be governed and carried out. However, decisions regarding de-implementation are not only a matter of scientific evidence; the puzzle is far more complex with political, economic, and relational interests play a role. This project aims at exploring the governance of de-implementation of low-value practices from the perspectives of national and regional governments and senior management at provider organizations.

**Methods:**

Theories of complexity science and organizational alignment are used, and interviews are conducted with stakeholders involved in the governance of low-value practice de-implementation, including national and regional governments (focusing on two contrasting regions in Sweden) and senior management at provider organizations. In addition, an ongoing process for governing de-implementation in accordance with current recommendations is followed over an 18-month period to explore how governance is conducted in practice. A framework for the governance of de-implementation and policy suggestions will be developed to guide de-implementation governance.

**Discussion:**

This study contributes to knowledge about the governance of de-implementation of low-value care practices. The study provides rich empirical data from multiple system levels regarding how de-implementation of low-value practices is currently governed. The study also makes a theoretical contribution by applying the theories of complexity and organizational alignment, which may provide generalizable knowledge about the interplay between stakeholders across system levels and how and why certain factors influence the governance of de-implementation. The project employs a solution-oriented perspective by developing a framework for de-implementation of low-value practices and suggesting practical strategies to improve the governance of de-implementation. The framework and strategies can thereafter be evaluated for validity and impact in future studies.

Contribution to the literature

- The study contributes knowledge about the governance of de-implementation of low-value care practices, an aspect of the research–practice gap that has received little attention so far.
- The study contributes by applying a health care system perspective on the governance of de-implementation, which provides insights into how actions and decisions by multiple stakeholders at the national, regional, and organizational levels may influence professional behaviors in clinical practice.
- The study provides knowledge that is useful in practice by creating theory-based policy suggestions that will be developed in collaboration with key stakeholders with extensive practical experience in the governance of de-implementation.

## Background

The emergence of evidence-based medicine and its broader application as evidence-based practice (EBP) has popularized the notion that research findings should be more widely utilized in various practice settings, including health care. Research on implementation science was borne out of a desire to address problems associated with using research to achieve a more evidence-based health care practice. However, developing a more EBP may also require de-implementation (i.e., stopping practices that are not evidence-based, usually referred to as low-value care) [1]. There are several potential reasons for discontinuing practices: a more effective practice may have become available, clinical effectiveness may not be documented in clinical trials or the practice may be harmful or of little value to all patients or sub-groups of patients. However, de-implementation may also be relevant for practices that are effective but not sufficiently cost-effective; this often leads to partial withdrawal such that an intervention, service, or program is still offered to some patients under certain conditions [2]. The challenge of constrained health care budgets has increasingly forced decision-makers and practitioners to consider how to discontinue practices that are no longer regarded as cost-effective [3–6].

The use of low-value practices is common. It has been estimated that 12–15% of patients receive at least one low-value practice a year [7], and 72% of physicians in the US state that they prescribe unnecessary tests or procedures at least once a week [8]. The most common form of low-value care is most likely the inappropriate use of an effective practice for patients for whom benefit has never been demonstrated [9]. The annual cost of low-value care was estimated to be $8.5 billion for the Medicare population in the USA, which is almost 3% of total annual Medicare spending on services [10]. However, despite the rapid growth of implementation science, strategies, and policies to achieve de-implementation thus far have attracted limited interest from researchers, professionals, and decision- and policy-makers [11, 12]. Thus there is insufficient knowledge about efficient governance of de-implementation, (i.e., translating *what* should be de-implemented into *how* this should actually be carried out and governed) [4, 9, 13–15]. From this follows that the lists of non-recommended practices that have become increasingly used, including Choosing Wisely [9] and the Swedish National Board of Health and Welfare’s not-to-do lists, have little chance of leading to improvements.

De-implementation has often been considered an individual issue (i.e., something that each health care professional makes decisions about). However, because multiple factors are likely to impact individuals’ decisions and behaviors, the challenge of de-implementation cannot only be considered health care professionals’ responsibility; rather, it is a health care *system issue* [16–18]. Among these factors are prevailing regulations, norms, values, work processes, and financial interests at the group, department, organization and state or national levels [4, 19]. Research on how these factors impact de-implementation and how these can be managed to obtain efficient de-implementation is limited, highlighting the need for a system-level approach to investigating the governance of de-implementation.

### Factors influencing the governance of de-implementation

There is a consensus among international researchers, policy-makers, and practitioners that, ideally, clinical effectiveness, cost-effectiveness, quality, and patient safety should determine decisions to de-implement a practice [12]. However, research has identified a gap between rhetoric and reality such that the ideal driving forces for de-implementation decisions differ from the factors that are most influential in real-world practice. Cost considerations tend to impact de-implementation decisions at the system level more than the clinical effectiveness of the practices [20]. Furthermore, political interests, relationships among stakeholders, vested interests, community and patient expectations, and media engagement often influence the success of de-implementation processes [12]. Thus, the governance of de-implementation appears to be complex and depends on many factors, with political, economic and relational interests playing important roles.

From the *perspective of administrative managers*, the decision to abandon low-value care often lacks a clear structure and offers few opportunities to monitor success [21]. For instance, administrative managers at the provider level in the UK have reported a lack of understanding and knowledge about how to efficiently de-implement low-value care. They have also perceived resistance and skepticism from clinicians regarding their decisions, which has contributed to making the process challenging [21]. Furthermore, available data on the outcomes of various care practices are often lacking or of poor quality, making it difficult to make qualified, well-informed decisions. A study of de-implementation practices among senior managers in Swedish health care systems confirms some of these findings, highlighting an urgent need to develop systematic de-implementation routines [22].

When exploring de-implementation from the *perspective of health care professionals*, the importance of governance and system issues becomes clear. Three key factors can be found in the literature connected to the health care system that influence professionals’ de-implementation decisions: (1) characteristics of the practice that is to be abandoned, (2) patient preferences concerning the practice, and (3) governance and organizational issues. The characteristics of the low-value practice concerned how clear the evidence for low-value care was and whether an alternative practice was available [20]. As regards patient preferences*,* patients’ wishes were a strong factor influencing physicians’ use of low-value care [23]. Concerning governance and organizational issues*,* the literature indicated the relevance of parallel steering mechanisms, including financial aspects, as important influences on whether practices are used or abandoned [8]. There appears to be a risk that line managers will encourage health care professionals to choose practices based on reimbursement levels rather than the scientific evidence for the practices. Furthermore, lack of transparency in de-implementation decision-making, lack of organizational systems to facilitate behavior change (e.g., lack of decision support systems), and poor communication and leadership in general negatively affected discontinuation of low-value care [12, 21, 24].

In sum, health care professionals’ practices are shaped and nuanced in complex ways by the wider system, health policies, knowledge-producing agencies, and existing power dynamics within the health care system. There is potential tension between health care professionals and administrative management regarding the credibility and legitimacy of de-implementation functions, reflecting the anxiety that economic issues, rather than scientific evidence, drive the process [21]. Furthermore, none of the countries where the studies were conducted (e.g., Sweden, UK) had efficient systems or governance for de-implementation of low-value care practices, making research into de-implementation both timely and important.

### Aim

This project aims to explore the governance of de-implementation of low-value practices from the perspectives of national and regional governments and senior management at provider organizations.

The following research questions (RQs) will be addressed:

1. How do actors involved in health care governance view their own and others’ roles, tasks, and possibilities to fulfill their roles and tasks in the de-implementation of low-value care practices?
2. Which factors at various governance levels impact de-implementation decisions (or lack of decisions) and processes?
3. How are professionals’ roles and autonomy considered in de-implementation decisions?
4. How can the governance process and factors that influence de-implementation governance be categorized and synthesized into a conceptual model?
5. How can de-implementation be governed across various health care system levels (national, regional, and organizational) to facilitate an effective process that also considers health care professionals’ autonomy?

### Theoretical approach

The project uses two theoretical approaches to explore the governance of de-implementing low-value care: complexity science and the theory of alignment. The starting point is in complexity science and the acknowledgment that systems function in a complex, non-linear manner in which structures, processes, and people are interdependent [25, 26]. Agents’ actions and systems such as health care [27] develop over time in a way that might seem unpredictable [28]. Agents such as health care professionals have their own mental models and sense-making processes, which are not necessarily shared or even logical when viewed by someone else in the system. One basic assumption is that actors in a system use their freedom to act and self-organize their actions [29]. They develop ways of functioning based on their perceptions of the most effective way of performing tasks, given their local context and resources. Applying complexity science to health care governance implies discarding the view that performance is optimized when work is planned, specified, and controlled in detail. Instead, a few flexible, simple rules could lead to more efficient governance with opportunities to take advantage of professionals’ competence, autonomy, and creativity [27].

The theory of alignment can be helpful when designing such governance mechanisms. This theory describes how organizational behaviors and practices are shaped by overarching priorities and goals [30, 31]. In an aligned organization, employees know what they are expected to do and how they can contribute to the goals of the organization [31]. Alignment implies processes and structures that give employees clear direction while providing opportunities for creativity and autonomy, which foster motivation. This implies that individuals’ opportunities, motivation, and ability to de-implement their work practices are dependent on the general expectations, norms, and values in the organizational context rather than on detailed steering of actions [32]. Thus, rather than trying to govern or micromanage behaviors in detail, the appropriate level of governance at various system levels is necessary [33]. A series of decisions determining the room for and desirability of variation at the lower levels of the system is needed. This does not imply a top-down governance approach. It is often employees, not managers, who have intimate knowledge of which behaviors drive key results. Nevertheless, general guidance at the right level of the system can provide direction, boundaries, resources, and permissions that, in turn, create an environment where innovative, shared actions can emerge [34]. Too little governance steering easily leads to chaos, but too much governance tends to cause frustration and avoidance of the regulation.

## Methods

This multi-disciplinary project starts with qualitative data collection to generate deeper insights into the phenomenon (steps 1, 2, and 3). Thereafter, we will develop a conceptual framework for the governance of de-implementation (step 4), which we will use to develop policy suggestions (step 5). The study will be conducted at the state and regional levels in the Swedish health care system.

### Data collection, analyses, and participants

#### Step 1

The first step involves *interviews with health care governance stakeholders* to answer RQ 1. This step also includes qualitative data collection, and the COREQ [35] checklist will be closely followed. Semi-structured interviews will be conducted with stakeholders at the national and regional governance levels (e.g., civil servants and managers) and with senior managers at provider organizations. Our overall goal is to understand how these stakeholders describe their own and others’ expected roles and tasks related to the governance of de-implementation of low-value care. Furthermore, our focus is on understanding their possibilities of fulfilling the roles and tasks in daily governance.

We will start with the stakeholders at the national level. Snowball sampling will be used, starting with recruiting individuals who are recognized as knowledgeable and experienced in the governance of de-implementing low-value practices. First, relevant organizations will be identified at the national level. These may not only include national organizations for knowledge governance (e.g., the National Board of Health and Welfare, Swedish Agency for Health Technology Assessment and Assessment of Social Services), but can also include other national organizations, such as professional associations, if these take an active role in influencing de-implementation processes. Individuals at each organization will be identified using the organization’s website or by asking representatives at the organization.

Thereafter, additional stakeholders will be identified at the regional level. Two regions will be selected with the purpose of including two contrasting cases based on the criteria that there are differences across these regions in regard to population, size, and the mode of governance of de-implementation of low-value practices at regional level or the organization of care at the provider level (e.g., a mixture of public and private). This includes the use of parallel governance strategies, such as financial reimbursement to provider organizations. The selection of contrasting cases will allow us to compare whether the governance of de-implementation of low-value care differs between the two regions, which increases the chances that the results will be transferable to other health care systems. A request to study the process will first be sent to the main stakeholders at the regional level. A snowball sampling will be used, starting with the individuals and groups recognized as most central to the regional process of interpreting the national guidelines and introducing them into local governance systems. Thereafter, relevant provider organizations will be identified, and representatives at senior management positions will be included to capture their perspectives on how these local governance systems are transferred to the level of provider organizations.

Thus, the respondents will be recruited to obtain a purposeful sample that is varied in terms of the participants’ function in the health care system and previous experiences with de-implementation governance. This diversity is sought to achieve richer variations of stakeholder perceptions and experiences regarding de-implementation. Approval for data collection will be sought from individuals recognized as central in the regional governance processes of interpreting and introducing national not-to-do lists to local governance systems. We will start with the individuals most involved in the specific de-implementation practice and thereafter identify other groups and individuals involved in the process. We will carefully explain that the study focuses on the process of de-implementing a practice rather than judging their use of this practice.

The interviews will focus partly on prior experiences with governing de-implementation of low-value care. We will also ask questions related to a current recommendation to de-implement a low-value practice. This recommendation will be selected from the National Board of Health and Welfare’s list of current recommendations. Recommendations with clear not-to-do lists that include low-value practices to be de-implemented will be selected, preferably from guidelines that include the de-implementation of both medical and psychosocial interventions. The practice to be studied will be selected based on, among other things, the strength and clarity of the scientific evidence for the low value of that particular care practice. Because the goal of the project is to explore the governance process of de-implementing a practice rather than potential discussions about the actual value of a practice, we will choose a practice with clear evidence for low value. The choice of low-value practices will guide the selection of provider organizations, as described above, focusing on organizations in which the recommendations of choice are highly relevant.

The semi-structured interviews will be based on an interview and informed by the guiding theories. The interviews will continue until saturation is achieved within each respondent group. We estimate that approximately 5–6 respondents in each group and region are needed, for a total of 40–50 interviews.

One member of the research team (SK) will be mainly responsible for data collection and analysis. The remaining researchers will act as informed outsiders. They will participate in iterative debriefing sessions to support the analysis and interpretation of the findings. Thus, data collection, transcription of recordings, and analysis will be performed iteratively. A deductive approach based on the guiding theories of complexity and organizational alignment will be used to analyze the data. Interviews will be recorded using a digital voice recorder and transcribed verbatim. NVivo will be used for the data analysis.

#### Step 2

This prospective step involves *longitudinal interviews* with a focus on the current de-implementation process to answer RQ 2. At this step, we follow the process of de-implementation at the national and regional levels in accordance with the current recommendation selected in step 1. The governance process for de-implementing the national-level recommendation and the consequent processes at the regional level will be studied over a period of 18 months with a focus on understanding which factors at various governance levels impact de-implementation decisions and processes. Thus, at this step, the project includes all governance actors involved in the interpretation of a particular not-to-do recommendation, including the identified provider organizations. Thus, the target group of step 2 mainly includes the organizations and individuals identified during step 1. However, if new actors or individuals who fit the purpose of the study are identified, they will be recruited for participation for this step. The number and timing of interviews will be determined during data collection.

The interviews in step 2 will focus on the process of the governance of de-implementation over time, and specifically on which factors at various governance levels impact the governance process. Throughout the interviews, multiple perspectives on the governance process will be explored, including the actors’ roles and responsibilities, as well as their views on how other actors influence the process. Particular attention will be paid to potential discrepancies (at multiple levels of governance) between what the selected recommendations state should be de-implemented, what is done in practice and the underlying reason behind such divergences. A multidimensional mode of analysis will be applied when analyzing the longitudinal data [36] to identify relevant factors influencing the governance process across various system levels and stakeholders, as well as analyzing the role of consistency and change over time. The iterative debriefing process used in step 1 will also be used here.

#### Step 3

To answer RQ 3, a focused analysis will be conducted that specifically addresses how actors at multiple governance levels consider health care professionals’ roles and autonomy in de-implementation decisions, including how these factors might change over time. Data used to answer RQ 3 will be collected as an integrated part of the data collection for steps 1 and 2.

#### Step 4

A conceptual model will be developed for the governance of de-implementation to answer RQ 4*.* During this step, we will synthetize the knowledge from the previous steps in a model for the governance of de-implementation of low-value care. The model will illustrate the governance process of de-implementation across various system levels and factors influencing the process. A comparison will also be made with one of the existing determinant frameworks for implementation. We have investigated numerous potential frameworks, many of which include the same or similar determinants [37]. We plan to use the EPIS (Exploration, Preparation, Implementation, Sustainment) framework [38] because it is one of the most comprehensive frameworks covering the implementation process, context, innovation, and practice factors potentially impacting the process. It is also sufficiently broad to allow for an explorative, inductive approach to collecting data. This approach is important because there are few studies which have investigated and attempted to describe the process of and categorize influences on the governance of de-implementation of low-value care.

A preliminary version will be iteratively tested and developed with stakeholders involved in health care governance and other researchers in scientific conferences to attain optimal scientific rigor and practical usefulness. The conceptual model is an important part of this work because it gives an overview of the areas covered by the policy solutions. During step 4, the focus will therefore gradually shift from an intensive focus on how de-implementation is conducted to potential solutions to improve the process.

#### Step 5

To answer RQ 5, policy suggestions for governing the de-implementation of low-value care will be developed based on the framework developed in step 4. Thus, the knowledge gained about the process and what impacts the governance of de-implementation will be put into practical suggestions that can be valuable to key actors involved in the governance of de-implementation of low-value care. The conceptual model will guide us in choosing suggestions that have the greatest impact on de-implementation processes at all levels in the health care system while considering professionals’ competence and autonomy.

A structured process, the co-created program logic (COP), will be used [39] to apply knowledge and experience from multiple sources to create a shared understanding among participants. COP is flexible in terms of number of participants; the final number will be decided based on the number of relevant participants who can be identified. Approximately 3–6 workshops will be conducted, and the various actors involved in health care governance at the national and regional levels will be invited. The stakeholders will be asked to rate the feasibility (e.g., applicability, comprehension, strengths, and weaknesses) of each strategy. Descriptive statistics will be used to analyze the data.

## Discussion

This study has an ambition to contribute to knowledge about the governance of de-implementation of low-value care practices. It will offer a wider contribution to the research on health care governance and implementation in terms of closing the well-known gap between research and practice. More specifically, the study makes five main contributions to the research on implementation and health care governance. First, it contributes via empirical data on how de-implementation of low-value practices is currently governed. De-implementation is an aspect of the research–practice gap that has received little attention and is considered something of an Achilles’ heel for health care systems. By offering insights into how actors governing health care make decisions and handle de-implementation in daily practice, this project constitutes an important starting point for research on the governance of de-implementation of low-value care. Furthermore, the empirical data concern several governance stakeholders at multiple system levels. Thus, the current study contributes a broad understanding of de-implementation of low-value practices in the health care system.

Second, the health care system perspective on the de-implementation of low-value care is highlighted. Abandoning certain work practices might require decisions and actions at organizational, regional, state, or national levels. The project will contribute to knowledge of how de-implementation can be understood across health care system levels, which is vital to ensuring that effective processes are achieved across the whole system. The contextual perspective on implementing new knowledge has been emphasized as crucial [40], which also illustrates the high value of the organization and system perspective.

The study’s use of complexity science and theory of alignment makes a novel and third contribution to the research of health care governance and implementation. These theories have been suggested as valuable [27], but few studies have been conducted. The theoretical approach can offer generalizable knowledge on how system levels interact in the governance of de-implementing low-value care practices. The study’s theoretical approach also contributes to understanding how and why certain factors impact the governance of low-value care practices and the de-implementation of low-value care, rather than merely listing which factors may have an impact. Fourth, the project will develop a conceptual model for the governance of de-implementation of low-value practices. This can be used as a framework in future studies that aim to approach de-implementation systematically. The conceptual model will illustrate various levels of the health care governance system (national, regional, and organizational) in relation to abandonment of low-value care practices.

Fifth, theory-based, practical policy suggestions will be made to improve de-implementation governance in the health care system. Because de-implementation and, more specifically, the governance of de-implementation is an under-researched area, there are few solutions available for researchers and practitioners. The policy suggestions will be tested for feasibility, and this project will offer the research community the opportunity to evaluate the impact of these solutions on de-implementation processes in future studies.

## Data Availability

The datasets used will be available from the corresponding author on reasonable request.

## Abbreviations

COP: Co-created program logic
EBP: Evidence-based practice
